# Anti-topoisomerase 1 Antibody Level Changes after B Cell Depletion Therapy in Systemic Sclerosis

**DOI:** 10.1134/S1607672923700266

**Published:** 2023-10-13

**Authors:** L. P. Ananyeva, L. A. Garzanova, O. A. Koneva, M. N. Starovoytova, O. V. Desinova, O. B. Ovsyannikova, R. U. Shayakhmetova, M. V. Cherkasova, A. P. Aleksankin, E. L. Nasonov

**Affiliations:** 1 Nasonova Research Institute of Rheumatology, Moscow, Russia; 2 Sechenov First Moscow State Medical University of the Ministry of Health Care of the Russian Federation (Sechenov University), Moscow, Russia

**Keywords:** systemic sclerosis, antinuclear antibody, anti-topoisomerase 1 antibody, interstitial lung disease, rituximab

## Abstract

The aim of our study was to assess the relationship between the changes of antinuclear autoantibodies (ANAs) and autoantibodies to topoisomerase 1 (anti-Topo 1) in systemic sclerosis (SSs) patients on rituximab (RTX) therapy. The prospective study included 88 patients (73 women) with a mean age of 47 (17–71) years. The mean disease duration was 5.9 ± 4.8 years. The mean follow-up period was more than 2 years (27 (12–42) months). We documented a statistically significant change in skin score, the disease activity index, improvement of pulmonary function and reduction of mean dose of prednisolone after RTX treatment. There was a significant decrease in the number of patients with high levels of ANA and overall decrease of the ANA and anti-Topo 1 levels. A moderate positive statistically significant correlation was found between ANA and anti-Topo 1 (*r* = 0.403). In the group of patients positive for anti-Topo 1 there were a more pronounced depletion of B lymphocytes, significantly higher increase in forced vital capacity and diffusion capacity, decrease in the disease activity index, compared with patients negative for anti-Topo 1. We observed the decline in the level of ANA and anti-Topo 1 in SSc patients after RTX therapy, and it was correlated by an improvement of the main outcome parameters of the disease. Therefore, anti-Topo 1 positivity could be considered as a predictor of a better response to RTX treatment, especially in SSc patients with hyperproduction of anti-Topo 1.

Systemic sclerosis (SSc) is a systemic immune-inflammatory (autoimmune) rheumatic disease (IIRD), the pathogenesis of which is based on immune disorders combined with vasospastic vascular reactions and leading to activation of fibrosis and uncontrolled deposition of extracellular matrix components in tissues [1]. SSc, as well as other IIRDs, is characterized by overproduction of autoantibodies to a wide range of nuclear and cytoplasmic molecules (ANAs, antinuclear autoantibodies), which are determined by the conventional methods such as indirect immunofluorescence analysis, as well as enzyme immunoassay, immunoblotting, etc. [2–4]. Along with the antibodies to the centromere (ACA) and RNA polymerase III, the antibodies that are specific for SSc include antibodies to the non-histone chromosomal protein Scl-70, which is the enzyme topoisomerase I with a molecular weight of 70 kDa (anti-Topo 1). In SSc, the detection of “sclerodermal” antibodies not only has a diagnostic significance (within the classification criteria for SSc) [5], but also makes it possible to identify the clinical and immunological subtypes that are characterized by different range of lesions of internal organs and determine the prognosis of the disease [6]. Anti-Topo 1, which are detected in one-third of SSc patients, is associated with the development of rapidly progressive skin fibrosis, interstitial lung disease (ILD), digital ulcers, and high mortality [7–9].

According to modern concepts, the disturbance of B-cell tolerance plays a fundamental role in the immunopathogenesis of SSc and other IIRDs [10, 11]. However, the specific mechanisms underlying the hyperproduction of anti-Topo 1 and their pathogenetic significance (as well as the autoantibodies detected in other IIRDs) are not completely clear [12]. The following facts testify to the potential pathogenetic significance of the immune response to Topo 1. According to experimental data, immunization of mice with Topo 1 induces the synthesis of anti-Topo 1 and the development of skin and lung fibrosis [13, 14]. In the lung tissue of SSc patients, an increase in the expression of Topo 1 was detected [15], and in the blood of patients with ILD, an increase in the number of autoreactive CD4+ T cells, which are specific for Topo 1 and have the Th17 “proinflammatory” phenotype, was noted [16]. In some SSc patients, decreased levels of anti-Topo 1 during treatment are associated with a milder course of the disease [17, 18]. However, according to other studies, the dynamics of the level of anti-Topo 1 during therapy, including after transplantation of autologous hematopoietic stem cells, is insignificant or absent [19–21].

A promising direction in the treatment of SSc is associated with anti-B-cell therapy with rituximab (RTX), which is chimeric monoclonal antibodies to B-cell CD20 [22, 23]. The clinical efficacy of RTX in SSc was demonstrated in many studies [24–29]. Preliminary results indicate that a decrease in ANA titers during RTX treatment in SSc patients is associated with a positive dynamics of skin count [30, 31]. In SSc patients in whose sera anti-Topo 1 was initially detected, the effectiveness of therapy with RTX was higher than that with cyclophosphamide [32].

All above served as a basis for a study aimed at investigating the relationship between the dynamics of the levels of antinuclear autoantibodies and autoantibodies to topoisomerase I in patients with systemic scleroderma and the effectiveness of rituximab therapy.

## MATERIALS AND METHODS

The study included 88 patients (age, 17 to 71 years; disease duration, 1 to 30 years) with a reliable diagnosis of SSc according to the criteria of ACR/EULAR (American College of Rheumatology/European League Against Rheumatism) of 2013 [5], who were treated with RTX (Table 1).

**Table 1.** Characteristics of patients included in the study (*n* = 88)

Parameters	Value
Age, years, *M* ± σ	47 ± 13
Sex, *n* (%)	
– female	73 (83)
– male	15 (17)
Disease subset, *n* (%)	
– limited	30 (34)
– diffuse	50 (57)
– overlap	8 (9)
Interstitial lung disease, *n* (%)	70 (80)
Disease duration, years, *M* ± σ	5.9 ± 4.8
Follow up, month, *M* ± σ	26.3 ± 10.7
Prednisolone mg/day, *M* ± σ	11.7 ± 4.4
Immunosupressants at baseline, *n* (%)	37 (42)
Cumulative mean dose of RTX (gr), *M* ± σ	2.9 ± 1.1
ANA HEp-2, *n* (%)	88 (100)
A-Topo-1 positivity, *n* (%)	63 (75)
ACA positivity, *n* (%)	3 (3.4)

RTX, rituximab; ANA, antinuclear antibody; A-Topo-1, anti-topoisomerase 1 antibody; ACA, anti-centromere antibody.

The basis for prescribing RTX was the severe course of the disease, the presence of unfavorable prognosis factors, or the insufficient effectiveness of standard therapy [26, 33]. To assess the effectiveness of RTX therapy, along with the assessment of the main parameters characterizing the activity of the disease [34], we determined the skin score [35], forced vital capacity (FVC) and diffusion lung capacity (DLCO) using spirometry (Master Screen PFT, Viasys, Germany). The results of functional pulmonary tests are given as a percentage of the expected values. Values of 80–120% of the due value were taken as the norm for both FVC and DLCO. Interstitial pneumonia was diagnosed on the basis of data from multislice computed tomography of the chest organs (MSCT of the chest). ANA was determined by indirect immunofluorescence using Hep-2 cells (Immco, United States). ANA titers ≤1 : 160 were taken as the upper limit of the norm. Anti-Topo 1 and ACA were detected by enzyme-linked immunosorbent assay (ORGENTEC Diagnostika, Germany). The upper limit of the norm for anti-Topo 1 was 25 U/mL; for ACA, 10.0 U/mL (according to the manufacturer’s instructions). The number of CD19+ B cells in peripheral blood was determined by flow cytometry (Cytomics FC 500 analyzer, Beckman Coulter, United States). The normal level of cells in the peripheral blood was 6–19%, 0.1– 0.5 × 10^9^/L. A complete depletion of CD19+ B cells was considered a decrease in their absolute number in the blood to a level of ≤0.005 × 10^9^/L. The results of the study were processed using the Statistica 10.0 statistical software package (StatSoft Inc., United States). To analyze the statistical significance of differences in parametric indices at a normal distribution of the studied parameter, Student’s *t* test was used. Differences were considered statistically significant at *p* < 0.05.

## RESULTS AND DISCUSSION

During therapy, a significant improvement in the main clinical parameters of the disease was observed (Table 2). The severity of dermal fibrosis (skin score) and the disease activity index decreased statistically significantly, lung function parameters improved, and the daily dose of prednisolone was reduced.

**Table 2.** Changes of clinical parameters of the disease during RTX (*n* = 88)

Parameters	Before RTX therapy	During RTX therapy	*р*
Rodnan skin score, *M* ± σ	11.21 ± 9.33	6.19 ± 4.74	0.001
Activity index (EScSG-AI), *M* ± σ	2.9 ± 1.74	1.36 ± 1.15	0.001
FCV (% predicted), *M* ± σ	76.35 ± 19.65	84.37 ± 21.04	0.001
DLCO (% predicted), *M* ± σ	45.56 ± 17.72	47.62 ± 16.96	0.019
B-lymphocytes (absolute count) (×10^9^/L), *M* ± σ	0.224 ± 0.19	0.0175 ± 0.058	0.001
Prednisolone (mg/day), *M* ± σ	11.7 ± 4.4	9.2 ± 3.2	0.001

RTX, rituximab; FVC, forced vital capacity % predicted; DLCO, diffusion capacity for carbon monoxide % predicted.

**Fig. 1.**
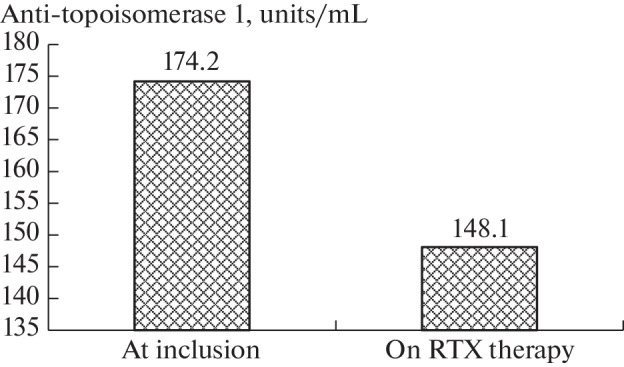
Changes of anti-topoisomerase 1 during rituximab (RTX) therapy (*n* = 63).

During RTX treatment, the number of patients with high ANA titers decreased. For example, before treatment, low ANA titers (from 1/160 to 1/320) were detected in 18 patients; high (≥1/640), in 70 patients. After treatment, the number of patients with low titers almost doubled (to 47), and the number of patients with high titers decreased to 41 (*p* = 0.00001). Simultaneously with the decrease in ANA titers (data not shown), in patients positive for anti-Topo 1, a decrease in the concentration of these antibodies from 174.2 ± 50.1 to 148.1 ± 66.1 U/mL (*p* = 0.0009) was detected (Fig. 1). Of the 63 patients initially positive for anti-Topo 1, the level of these antibodies decreased to normal values in 5 (7.9%) patients. The absence of anti-Topo 1 was accompanied by a clear improvement in the skin score (–7.4 points). A decrease in ANA titers correlated with a decrease in the severity of dermal fibrosis (skin score reduction) (*r* = 0.26; *p* = 0.014).

Data regarding the effectiveness of RTX therapy depending on the initial detection of anti-Topo 1 are presented in Table 3. As can be seen from Table 3, the effectiveness of RTX therapy in terms of the dynamics of the activity index, lung function, and skin lesions in the group of anti-Topo 1-positive patients was statistically significantly higher than in anti-Topo 1-negative patients.

**Table 3.** Changes (Δ) of the main outcome parameters depending on the presence of anti-topoisomerase 1 (a-Topo-1) on rituximab (RTX) therapy (*n* = 88)

Parameters	a-Topo-1 positive (*n* = 63)	a-Topo-1 negative (*n =* 25)	*p*
Δ Activity index (EScSG-AI)	1.79	0.9	0.001
Δ Rodnan skin score	4.9	5.2	NS
Δ FVC, % predicted	8.64	6.46	0.001
Δ DLCO, % predicted	2.86	0.032	0.001

NS, nonsignificant; FVC, forced vital capacity, % predicted; DLCO, diffusion capacity for carbon monoxide, % predicted.

The obtained data supplement and expand the results of our previous studies [25, 26] and studies of other authors [27, 28, 36–38], indicating the effectiveness of RTX in relation to the overall activity of SSc, as well as skin and lung fibrosis. Of particular interest are data on the decrease in the level of anti-Topo 1 and a higher efficacy of RTX in the patients with anti-Topo 1-positive SSc subtype. This is somewhat consistent with the data on the effects of RTX in other IIRDs. For example, in patients with systemic lupus erythematosus, treatment with RTX leads to a decrease in the titers of antibodies to double-stranded DNA, antibodies to cardiolipin [39–41], and antibodies to C1q [42]. In patients with rheumatoid arthritis (RA), treatment with RTX led to a decrease in the titers of rheumatoid factors (RFs), antibodies to vimentin, and, to a lesser extent, antibodies to cyclic citrullinated proteins (ACCPs) [43–45]. Similar results on the dynamics of antineutrophil cytoplasmic antibodies were obtained in patients with systemic vasculitis [46, 47]; antibodies to the glomerular basement membrane, in patients with Goodpasture’s syndrome [48, 49]; antibodies to the phospholipase A2 receptor, in patients with membranous nephropathy [50, 51]; antibodies to platelets, in patients with immune thrombocytopenia [52]; antibodies to erythrocytes, in patients with autoimmune hemolytic anemia [53]; antibodies to pancreatic islet cells, in patients with diabetes mellitus [54]. In RA, a high basal level of RFs and ACCPs is associated with the effectiveness of RTX therapy [55, 56]. In patients with immune thrombocytopenia, the absence of antibodies to platelets correlated with resistance to RTX therapy [57], and ANA positivity, on the contrary, was a predictor of a good response to RTX [58]. The clinical efficacy of RTX therapy in IIRDs in general and SSc in particular and the decrease in autoantibody titers correlate with the severity of B-cell depletion [59], which is consistent with our results. According to the literature, in patients with diffuse SSc who were treated with RTX for 5 years, an increase in FVC and a decrease in skin score correlated with a decrease in ANA and anti-Topo 1 titers [30]. Temporary cancellation of RTX led to an increase in the level of anti-Topo 1 and the severity of skin fibrosis, and the resumption of therapy led to a positive dynamics in the clinical manifestations of SSc. In another study, SSc patients treated with RTX showed a decrease in the level of ANA and “sclerodermal” autoantibodies, which correlated with the positive dynamics of SSc activity and a decrease in skin fibrosis [31]. It should be emphasized that, during treatment with RTX, a decrease in anti-Topo 1 titers is not associated with a simultaneous decrease in the concentration of IgG and IgG antibodies to the Epstein–Barr virus [31]. This fact indicates the relative specificity of the RTX effect in relation to the suppression of autoantibody synthesis. Important results were obtained by Boonstra et al. [60], who found an association between the SSc progression and an increase in the concentration of anti-Topo 1 of both IgG and IgM isotypes, whereas the detection of only IgG anti-Topo 1 did not correlate with the course of the disease. It should be noted that IgG anti-Topo 1 titers were stable and did not depend on SSc progression, whereas IgM anti-Topo 1 titers significantly fluctuated both upwards and downwards. This may reflect the development of two types of immune response to Topo 1 in SSc. One type is associated with T-cell-dependent activation of long-lived plasma cells (PCs) that synthesize IgG anti-Topo 1 in the absence of additional antigenic stimuli. The second type, which depends on the permanent activation of Toll-like receptors in short-lived plasma cells synthesizing IgM anti-Topo 1, more adequately reflects the current immune-inflammatory process. However, the nature of external stimuli that induce the activation of Toll-like receptors is not clear and requires special study. On the basis on these data, it can be assumed that the point of application of RTX in SSc is a subpopulation of autologous autoantibody-synthesizing short-lived B cells with the CD20+ CD19^med+^ IgD-CD27^hi^ CD39^hi^ phenotype and/or activated switch memory B cells (CD19+ IgD-CD27+ CD38-CD95+) [61], which are “sensitive” to RTX depletion [62]. It is noteworthy that an increase in the level of these B cells in the peripheral blood is statistically significantly associated with a high concentration of anti-Topo 1 and the development of pulmonary fibrosis [61]. Another potential mechanism of action of RTX may be associated with the suppression of the T-cell immune response. The level of CD4+ T cells autoreactive to Topo 1 is higher in the SSc patients whose sera were found to contain anti-Topo 1 than in the patients with anti-Topo 1 negative results [16]. It should be noted that Topo 1+ T cells have a Th17 “proinflammatory” phenotype, and an increase in their level is associated with the development of ILD and a decrease in FVC and DLCO. In this regard, it is of interest that, in IIRDs (and, in particular, in RA), CD20 expression is observed not only on B but also on T [63] and Th17 [64] cells and that RTX induces depletion of T [63] and Th17 [65] cells and a decrease in the Th17cell immune response, which manifests itself in the synthesis of interleukin (IL) 22 and IL-17 [65]. Finally, since the synthesis of IL-6 by Topo 1-specific T cells in SSc patients is of key importance for the effective activation and production of anti-Topo 1 by autologous B cells [66], it can be assumed that a decrease in the level of anti-Topo 1 during RTX treatment may be associated with depletion of B cells synthesizing IL-6 [67, 68]. It should be recalled that IL-6 exhibits a pronounced profibrotic activity [69], and its inhibition is considered a promising method of therapy for SSc [70, 71].

Thus, the use of RTX in SSc patients leads to a decrease in ANA and anti-Topo 1 titers, which is associated with the clinical effectiveness of therapy. It can be assumed that RTX therapy may be especially relevant for the SSc subtype associated with overproduction of anti-Topo 1.
