# Erratum to: **Anti-topoisomerase 1 Antibody Level Changes after B Cell Depletion Therapy in Systemic Sclerosis**

**DOI:** 10.1134/S1607672923050022

**Published:** 2024-01-24

**Authors:** L. P. Ananyeva, L. A. Garzanova, O. A. Koneva, M. N. Starovoytova, O. V. Desinova, O. B. Ovsyannikova, R. U. Shayakhmetova, M. V. Cherkasova, A. P. Aleksankin, E. L. Nasonov

**Affiliations:** 1 Nasonova Research Institute of Rheumatology, Moscow, Russia; 2 Sechenov First Moscow State Medical University of the Ministry of Health Care of the Russian Federation (Sechenov University), Moscow, Russia

The article “Anti-topoisomerase 1 Antibody Level Changes after B Cell Depletion Therapy in Systemic Sclerosis”, written by L.P. Ananyeva, LA. Garzanova, O.A. Koneva, M.N. Starovoytova, O.V. Desinova, O.B. Ovsyannikova, R.U. Shayakhmetova, M.V. Cherkasova, A.P. Aleksankin, and E.L. Nasonov, was originally published electronically in Springer-Link on October 13, 2023 without Open Access. After publication in volume 511, pages 212–218, the authors decided to make the article an Open Access publication. Therefore, the copyright of the article has been changed to © The Author(s) 2023 and the article is forthwith distributed under the terms of a Creative Commons Attribution 4.0 International License (http://creativecommons.org/licenses/by/4.0/, CC BY), which permits use, duplication, adaptation, distribution and reproduction of a work in any medium or format, as long as you cite the original author(s) and publication source, provide a link to the Creative Commons license, and indicate if changes were made.

